# Cereal-Derived Water-Unextractable Arabinoxylans: Structure Feature, Effects on Baking Products and Human Health

**DOI:** 10.3390/foods13152369

**Published:** 2024-07-26

**Authors:** Manchun Huang, Juan Bai, Daniele Giuseppe Buccato, Jiayan Zhang, Yufeng He, Ying Zhu, Zihan Yang, Xiang Xiao, Maria Daglia

**Affiliations:** 1 School of Food and Biological Engineering, Jiangsu University, Zhenjiang 212013, China; manchel2019@outlook.com (M.H.); 1000005134@ujs.edu.cn (J.B.); jiayanzhang1988@163.com (J.Z.); hyf_fbs@foxmail.com (Y.H.); ying307@126.com (Y.Z.); yzhyhjx@163.com (Z.Y.); 2 Department of Pharmacy, University of Napoli Federico II, Via D. Montesano 49, 80131 Naples, Italy; danielegiuseppe.buccato@unina.it; 3 International Research Center for Food Nutrition and Safety, Jiangsu University, Zhenjiang 212013, China

**Keywords:** water-unextractable arabinoxylans, structure feature, health benefits, gut microbiota, fermentation

## Abstract

Arabinoxylans (AXs) are non-starch polysaccharides with complex structures naturally occurring in grains (i.e., barley, corn, and others), providing many health benefits, especially as prebiotics. AXs can be classified as water-extractable (WEAX) and water-unextractable (WUAX) based on their solubility, with properties influenced by grain sources and extraction methods. Numerous studies show that AXs exert an important health impact, including glucose and lipid metabolism regulation and immune system enhancement, which is induced by the interactions between AXs and the gut microbiota. Recent research underscores the dependence of AX physiological effects on structure, advocating for a deeper understanding of structure-activity relationships. While systematic studies on WEAX are prevalent, knowledge gaps persist regarding WUAX, despite its higher grain abundance. Thus, this review reports recent data on WUAX structural properties (chemical structure, branching, and MW) in cereals under different treatments. It discusses WUAX applications in baking and the benefits deriving from gut fermentation.

## 1. Introduction

The global market has seen a significant increase in the number of whole-grain products in recent decades [1]. These products have the potential to reduce the risk of cardiovascular disease, diabetes, and gastrointestinal cancers by lowering cholesterol and lipid accumulation, improving insulin resistance, and promoting a healthy gut microbiota ecosystem [2,3,4,5,6,7,8,9,10]. The dietary fiber found in cereals, specifically arabinoxylan (AX), plays a crucial role in reducing the risk of diet-related chronic diseases. AXs are a non-starch polysaccharide commonly found in cereals such as corn, wheat, barley, oat, and sorghum [11,12,13,14]. Typically, AXs consist of a linear backbone of 1,4-linked β-D-xylopyranosyl (1,4-β-D-xylp) residues and α-L-arabinofuranosyl (α-L-araf) units that can be substituted at the C(O)-2 and/or C(O)-3 positions [15,16]. In addition, the xylose (xyl) unit can carry methyl glucuronide, and the arabinose unit can be bonded to ferulic acid residues [17]. Also, both xylose and arabinose can be acetylated. However, the structure of AX varies between different grains and even within the same grain [18].

The extraction of AX from grains can be performed using various methods, each resulting in unique structural features [19,20,21]. Nonetheless, the structure of AXs obtained from different raw materials or different extraction methods is not identical. These structural characteristics, such as the content of ferulic acid, molecular weight (MW), and substitution, influence the functional properties and physiological activities of AXs [22].

There are two types of AXs based on solubility: water-extractable arabinoxylans (WEAX) and water-unextractable (WUAX). WEAX is a small fraction of total AXs and is loosely bound to the cell wall surface, while WUAX is the most abundant fraction and is connected to other cell wall components [23,24,25,26]. WUAX, present in the cell wall and bound to other components like proteins, lignin, and cellulose through covalent and non-covalent bonds, requires the use of alkaline solutions to dissolve these bonds.

In recent years, AXs have found applications in the baking industry as they interact with other components of flour and dough to enhance the quality of bread. AXs interact with gluten, which has been recognized as one of the main components controlling the quality of dough and bread [27]. It has been proven that the amounts of WEAX and WUAX influence the rheological behavior of dough and the quality characteristics of bread [28]. WEAX and WUAX have different effects on baking quality; however, the presence of WUAX can negatively impact baking quality [29].

The gut microbiota has received increased attention and is considered an eminent symbiotic partner in the maintenance of human health [30]. The gut microbiota can define and regulate host homeostasis by determining nutritional, immune, and neuroendocrine homeostasis [31]. AXs also play a crucial role in regulating the composition of the gut microbiota and promoting intestinal health [32], with a variety of bioactivities such as immune and antioxidant enhancement, strengthening of the intestinal epithelial barrier, relief of constipation, and improvement of lipid and glucose metabolism [5,12]. AXs can modulate the profile and function of some of the beneficial bacteria in the human gut microbiota [33]. The structural properties of AXs determine their biological effects, fermentation process, and regulation of the intestinal microbiota. The MW of WEAX has been found to influence its prebiotic efficiency [34].

While most AXs in cereals are WUAX, these have not received as much attention as WEAX. Therefore, this paper aims to study the structure–activity relationship and treatment of WUAX in cereals, as well as its applications in baking and potential functional benefits for human health.

## 2. Structural Features

### 2.1. Monosaccharide Compositions and Branching Degree

**Figure 1:**
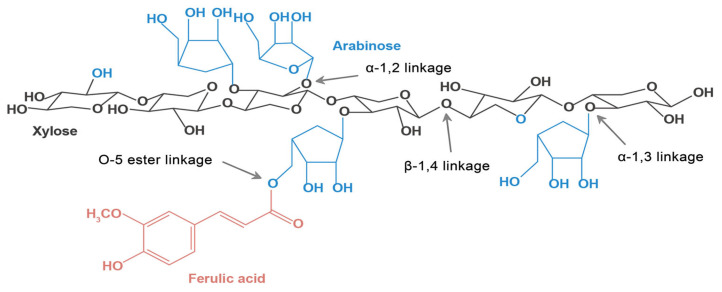
Structure of arabinoxylan, showing the xylose backbone (black), arabinose substitutions (blue), ferulic acid (red). The main chain of xylose is connected by the β-1,4-glucoside bond, arabinose can be linked to the main chain by the α-1,2 or α-1,3-glucoside bond, and ferulic acid is substituted at the O-5 position of arabinose by the ester bond.

AX is composed of a xylose backbone with arabinose substitutions and ferulic acid, connected by various types of glycosidic bonds (Figure 1).

The monosaccharide composition and the ratio of arabinose to xylose (A/X) describe the degree of branching in AX. A lower A/X indicates less branching. Although of different origins, most AXs comprise arabinose, xylose, glucose, and galactose [35]. While α-L-arabinofuranosyl is the main substitution on xylan backbones, it has been shown that small amounts of other sugar residues (i.e., glucuronic acid, 4-O-methylglucuronic acid (MeGlcA), or short oligomers consisting of L-arabinose, D-xylose, D-galactose, D-glucose, and/or glucuronic acid) can act as substituents [36]. The monosaccharide composition of WUAX from spelt bran extraction was arabinose, xylose, mannose, glucose, and galactose, with xylose having the highest percentage [37]. The pattern and extent of arabinose substitution vary across plant species and tissue locations [38]. Arabinogalactan and glucomannan are potential sources of galactose or mannose [39]. The monosaccharide composition of AX in cereals generally has a higher proportion of arabinose and xylose. Based on data obtained by alkali extraction of WUAX from different cereals, wheat flour had 46.6% arabinose and 48.6% Xyl of AX [40], and rye bran had 36.53% arabinose and 63.31% Xyl of AX [41]. Research has shown that the proportion of Xyl in AX extracted from oats is as high as 78% [42]. WUAX has a higher polydispersity and a lower A/X compared to WEAX. In general, the A/X ratio for AX in cereals ranges from 0.5 to 1.0, with sorghum and rice reaching 1.0, wheat close to 1, and the remaining cereals such as barley (0.23–0.58), maize (0.34–0.56) and rye (0.46–0.60) being smaller [43,44]. The A/X ratio of WUAX in spelt bran was 0.60–0.65, and the A/X of wheat was in most cases greater, meaning that the arabinose branch in spelt bran WUAX is relatively small. There are higher levels of galactose in rice and barley bran compared to other cereals with AX [45,46], and there is no detectable mannose in AX from wheat and oats. The A/X ratio of WUAX in 12 corn bran samples extracted using the alkaline method by Zhang et al. was also relatively low, with a range of 0.46~0.54 [47]. Different grains have varying branch chain and monosaccharide compositions, with WUAX generally having a higher branch chain degree and lower arabinose substitution, resulting in high MW and low solubility in water. The differences in monosaccharide composition are typically associated with grain type and genotype.

In recent years, research on the structure of WUAX has not been systematically evaluated. Targeted and in-depth studies on different genotypes or structures of WUAX from specific cereal sources should be conducted to explore the correlation between its structure, functional activity, and physicochemical properties. This will greatly aid in understanding the conformational relationships of WUAX.

### 2.2. Glycosidic Linkages

Methylation analysis is commonly used to determine the glycosidic linkage of AX across different grain sources. In cereal AX, the 4-xylp backbone, composed of xylose sugar residues, is predominantly present with branching points at the 0–2 and/or 0–3 positions. Small amounts of txylp (terminal xylopyranosyl), 2-xylp, and 3-xylp can also be detected using this method [48,49]. Arabinose-based sugars, including t-araf and 3-araf, are detected in all cereal sources, while other arabinose residues, such as 2-araf, 4-araf, 5-araf, 2,5-araf, and 3,5-araf, are only occasionally detected in cereals. The linear backbone of AX consists of D-xylopyranose linked by β-1,4 glycosidic linkages. α-L-furan arabinose can be monosubstituted or disubstituted at the O-2 and O-3 positions, and ferulic acid and 4-O-methylglucuronide can also act as substituents [50]. Ferulic acid, when attached to arabinose residues via ester linkages at the C(O)-5 position, can have significant effects on the functional properties of AX. Xylp residues form the backbone through 1→4 linkages and occur in three forms: unsubstituted, monosubstituted, and disubstituted. Arabinofuranosyl molecules are mainly present as monosubstituted residues at O-3. A study by Zhang et al. found that the side chain of the arabinose β-furanosyl residue in maize bran AX was mono-linked at the O-3 position. Xylopyranosyl groups with different substitution patterns have different A/X ratios, which carry important implications for the structural features and biological activities of AX [47]. The methylation analysis by Guo et al. showed that the xylose-based sugar residues in WUAX from alkali-extracted wheat bran contain T-xylp, 4-xylp, 3,4-xylop, and the arabinose-based sugar residues contain t-araf, 3-araf, and 2,3-araf. The xylan backbone contains 46.72% unsubstituted xylp, 27.42% monosubstituted xylp at the 0–3 position, and 25.88% doubly substituted xylp at the 0–2 and 0–3 positions [51]. Guo et al. extracted a relatively low-branched AX from hulled barley, and of this, 71.19% of unsubstituted (1,4-linked β-D-xylan), 14.78% of mono-substituted (1,3,4-linked β-D-xylan), and 10.76% of doubly substituted (1,2,3,4-linked β-D-xylp) xylose units were backbone components via β-(1→4) bonds [46]. However, it has also been shown that the AX of isolated barley and rye contained more AX molecules monosubstituted at O-2 [24,52]. Mono-substituted and di-substituted WUAX are present not only in hulled barley bran but also in wheat flour and rye bran [43]. These studies have confirmed that both monosubstituted and disubstituted WUAX can be present in cereals. This difference also affects the glycosidic bond composition of AX and implies that the structure of AX is more heterogeneous and different.

### 2.3. Combination of Phenolic Compounds

Grains contain high levels of phenolic compounds (e.g., ferulic, coumaric, p- coumaric, caffeic, chlorogenic, and eugenic acids) [53]. AXs can exhibit antioxidant activity by binding to these phenolic compounds. Xylan backbones with high substitution and low ferulic acid (FA) content are more water-soluble, while AX with low substitution and high ferulic acid content is water-insoluble [54]. Soluble dietary fibers, including β-1,3/1,4 glucan, water-soluble AX, and arabinogalactan protein, have low binding to phenolics (10%), whereas insoluble dietary fibers such as water-insoluble AX and cellulose-lignin complexes have high binding to phenolic acids (90%) [55]. The total phenolic acid content of WUAX obtained by Srinivasan et al. from alkali extracts of four millet varieties was 104.3, 164.6, 345.5, and 1307.7 μg/g, respectively, all containing p-coumaric acid and ferulic acid, with ferulic acid being the most represented, suggesting that the phenolic acid content of AX is highly correlated with genotype in addition to structure and extraction method [56,57].

Ferulic acid is the most prevalent and abundant phenolic compound that binds to AX, but alkali-extracted WUAX contains less ferulic acid because the hydrolysis of alkali extraction breaks the covalent ester bond linking ferulic acid to the AX residue. Nevertheless, a sequential process using subcritical water, membrane filtration, and selective enzyme treatment allowed some retention of ferulic acid in the AX, allowing the extraction of feruloyl AX and arabinoxylo-oligosaccharide (AXOS) from wheat bran. Bijalwan et al. determined the phenolic acids bound in hydroxycinnamic acid-bound arabinoxylan (HCA-AX) in five different millets by liquid chromatography-mass spectrometry (LC-MS) extraction, and the results showed that ferulic acid was the highest across all millets [58]. In a study by Ayala-Soto et al., various phenolic compounds such as ferulic acid, p-coumaric acid, and other hydroxycinnamic acids were detected in the products obtained from the alkaline extraction of WUAX from three different sorghums. The total hydroxycinnamic acid content ranged from 0.16 to 0.24 mg HCA/mg db (on a dry basis) and ferulic acid from 0.09 to 0.16 mg FA/mg db [59]. 

The binding of AX to phenolic acids significantly affects its functional activity, and different types of phenolic compounds have varying effects on its antioxidant capacity [60]. In a study by Li et al. the phenolic acid content was increased by binding ferulic acid more strongly to corn WUAX obtained by alkali extraction, and it was found that the structure of corn arabinoxylan (CAX) was not perturbed during the modification, with an A/X ratio of about 0.62 and an increase in the average MW accompanied by a significant increase in the antioxidant capacity [53]. Li et al. combined natural caffeic acid (CA) with maize WUAX obtained by alkali extraction through esterification to obtain corn bran arabinoxylan (CA-CAX) with varying degrees of substitution [61]. The results show that the antioxidant capacity of CA-CAX was significantly higher than that of CAX with increasing concentration and degree of substitution. Guo et al. found that the MW of AX decreased from 694 kDa to 386 kDa after grafting catechin onto alkali-extracted wheat bran WUAX in the presence of hydroxyl radicals, indicating that AX was degraded, but the linkage pattern was not significantly different from that of alkali-extracted AX [51]. Ayala-Soto et al. determined the phenolic acid content of AX in different fibers such as maize fiber (MF), insect-resistant maize pericarp (RP), and insect-susceptible maize pericarp (SP) and found that the antioxidant capacity of AX was related to the total phenolic, dehydrotriferulic acid (tri-FA), and p-coumaric acid (p-CA) concentrations, but differences in the A/X ratio of WUAX obtained from different fiber sources after alkali extraction were not significant [62].

As the phenolic acid content significantly influences the antioxidant and other functions of WUAX, researchers can explore various extraction methods to maintain or even enhance the phenolic acid content of WUAX. This will have great importance for the development of WUAX-based functional foods.

## 3. Effects of Different Treatments on WUAX Structure in Cereals

### 3.1. Alkali Extraction

**Figure 2:**
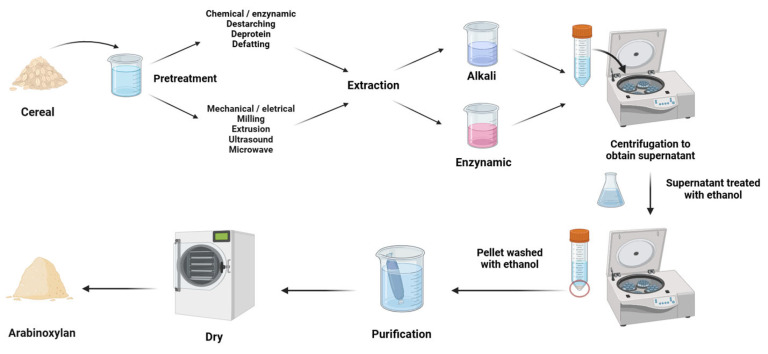
Chemical treatments for WUAX extraction.

Aqueous treatment can easily extract WEAX, which is not strongly bound to the cell wall surface of cereals. However, WUAX, present in the cell wall and bound to other components like proteins, lignin, and cellulose through covalent and non-covalent bonds, requires the use of alkaline solutions to dissolve these compounds. Alkaline extraction breaks covalent and hydrogen bonds in the polysaccharide matrix, releasing various polysaccharides from the cell [63,64,65,66]. Since the carboxyl group in AX can become negatively charged in an alkaline environment, leading to repulsion between different molecules, AX is easier to extract [67]. Therefore, alkaline extraction methods (NaOH, Ba(OH)_2_) are commonly used to extract WUAX. This extraction process is shown in Figure 2. 

The extraction of AX with different types of alkaline solutions also leads to different changes in the structure of AX. As the reaction during alkali extraction is very intense, it destroys functional groups such as ferulic acid [68]. Table 1 describes the effects of alkali extraction on the A/X ratio and MW of WUAX in different cereals. 

The degree of disruption of the arabinose substituents during alkaline treatment is not high, resulting in an increase in the A/X ratio [77]. Gruppen et al. found that the structure of AX obtained from wheat by alkali extraction (AE-AX) was almost identical to that of WEAX, with only minor differences in average MW, with AE-AX being higher than WEAX and with a slightly higher A/X ratio due to a higher proportion of arabinose side branches [78]. The A/X ratio of WUAX extracted from three different sorghum brans by an alkali method was higher, namely, white sorghum bran (1.41), which was higher than that of red sorghum bran (1.08) and high tannin bran (1.14) [59]. In addition to the typical structure of WEAX in specific grains, such as the A/X of WEAX extracted from wheat, which is 0.5–0.6, there are still great differences within different grains. For WUAX, in addition to differences between species, its structure is highly dependent on variables in the extraction process, such as the type of solvent used for alkali extraction and its concentration. WUAX obtained from wheat bran by alkali extraction had a MW of 694 kDa, and the A/X molar ratio of AX was 0.95, indicating a highly branched molecular structural formula [51]. Oat WUAX obtained by extraction with saturated Ba(OH)_2_ had very low substitution, whereas WUAX extracted with 1 or 6 M NaOH had small substitution branches and aggregated readily [42]. Furthermore, it was found that extraction with NaOH was also able to yield AX highly substituted with arabinose, with an A/X of 0.83 [69]. Guo et al. used a modified alkali extraction method to extract WUAX from hull-less barley bran, yielding HBAX-25, a relatively low-branched AX with an average MW of 298.36 kDa and an A/X of 0.58 [46]. Buksa et al. The MWs of WEAX and WUAX were compared in wheat, and it was found that the MW of WUAX is higher than that of WEAX, with the trend of AX in rye being almost identical to that in wheat [79].

In addition, WUAX obtained through alkali treatment typically has a high molecular weight, while the structural characteristics of AX (such as the A/X ratio) remain closely tied to the grain type. Even when extracted from the same grain using identical alkali solutions, AX can exhibit varying structures, likely influenced by factors such as alkali concentration and extraction conditions. However, the main factor influencing AX structure seems to be the grain variety and genotype.

### 3.2. Enzymatic Extraction

Enzymatic treatments are widely recognized for their ability to extract WUAX from cereals, breaking the link between AX and the cell wall and converting it to WEAX. AX can be degraded more accurately and efficiently using enzymatic extractions, which are more environmentally friendly and provide the same results as chemical methods such as alkaline extraction [80,81,82,83]. Additionally, enzymatic treatment can help retain phenolic substituents such as ferulic acid. Studies have shown that enzymatic extraction of WUAX using endo-xylanase is as effective as chemical methods [84]. Zhou et al. compared alkaline and enzymatic extraction of AXs from wheat bran and found that the enzymatically extracted AX had a lower average MW and polydispersity, lower substitution, and contained more protein and ferulic acid than the alkaline-extracted AX, which may induce a higher immune response [69]. A comparison of the structures of WEAX and XEAX (xylanase-extracted arabinoxylan) obtained by enzymatic digestion in wheat endosperm showed that XEAX has a different degree of substitution compared to WEAX and that XEAX has more monosubstitution and less unsubstitution than WEAX [85]. Ma et al. investigated the effect of dual enzymes on the extraction rate and AX content of extracts and confirmed that combined enzymatic extraction of fresh maize fiber using xylanase and cellulase was more effective than chemical methods [86]. 

The structure of AX obtained by enzymatic treatments depends on the specific enzyme used [87]. The main enzyme families involved in the degradation of AXs are endo-β-(1,4)-xylanase (EC 3.2.1.8), β-(1,4)-xylosidase (EC 3.2.1.37), α-L-arabinofuranosidase (EC 3.2.1.55), and ferulic esterase (EC 3.1.1.73) [88]. Endo-xylanases can attack the backbone of AX, changing its structure and affecting its function [89]. Enzymatic degradation of AX usually starts with the stripping of lignin from the cell wall, and since ferulic acid is the bridge between lignin and AX, the ester bond between arabinose and ferulic acid can be broken by ferulic acid esterase. Highly specific enzymes such as endo-xylanase and xylanase are used to convert WUAX to WEAX. However, endo-xylanases are not only able to convert WUAX to WEAX but also further degrade it into its individual components (arabinose and xylose) [64,90]. To further degrade AX, arabinofuranosidase can remove side chains from the xylose backbone, and xylanase can degrade the xylose backbone. 

Xylanases are the enzymes primarily involved in the degradation of AX and can be classified into two main families of glycoside hydrolases: glycoside hydrolase family 10 (GH10) and glycoside hydrolase family 11 (GH11). Most GH10 family xylanases can act on the xylosidic linkage at the non-reducing end of the substituted residue, resulting in a larger AXOS than that processed by GH10 xylanases due to the greater hindrance of the substituted group to GH11 xylanase [42,91]. This further suggests that GH11 has a higher substrate specificity than GH10. The polysaccharides in rice, wheat, and maize obtained by enzyme treatment were analyzed. It was found that the A/X ratio was less than 1, except for rice, which had a high A/X ratio (3.6) [55]. The MW of AX (51.6–132 kDa) obtained after treatment of WUAX in maize bran with xylan endonuclease by Kale et al. (2018) was lower than that of natural AX (253 kDa) [92]. This may be due to the destruction of the β-1,4-glucoside linkage of the xylose main chain by endo-xylanase while affecting arabinose substitution in the xylose chain. Table 2 describes the effects of two major enzymes on the A/X ratio and MW of WUAX in different cereals.

The method of enzyme preparation also has a specific effect on the structure of AX. Enzymatic extraction offers a promising alternative to traditional methods for extracting WUAX from cereals. The average MW of the alkali-extracted AXs was 3.5 × 10^2^ kDa, which was approximately 10 times higher than that of the enzymatically extracted AX (32.52 kDa) [69]. In addition, the concentration of an enzyme can modify the MW of AX; for example, in endo-xylanase the MW of AX decreased from 6.2 kDa to 2 kDa as the quantity of enzyme was increased [95]. Enzymatically extracted WUAX has a lower molecular weight and retains more phenolic acid compared to alkaline-extracted WUAX. Thus, it is important to understand the impact of the extraction conditions on the AX chemical structure, as if they are fully understood and utilized, they can contribute greatly to the specific extraction of WUAX with different structures.

### 3.3. Microbial Fermentation

Several studies on WUAX have indicated that the fermentation of grains can lead to structural changes and degradation. For example, during spontaneous fermentation of maize bran, the content of WEAX and free ferulic acid content increase, and the A/X ratio decreases [96]. An increase in insoluble polysaccharides (arabinose + 13%, xylose − 3%, and glucose + 53%) was observed in wheat bran after fermentation with *Lactobacillus plantarum* [97]. Fermentation of wheat bran by *Bacillus* spp. also leads to the appearance of different peaks of xylose and arabinose in the chromatogram of the extract [98]. 

Similarly, the degradation of WUAX can also occur during the production of grain products such as bread. Kiszonas et al. studied the changes in AX levels during bread-making and found that WEAX levels increased during dough fermentation regardless of whether whole wheat or refined flour was used [99]. This may be due to the hydrolysis of WUAX during this process. The chemical structure of AX changes during bread-making and dough fermentation. Analysis of samples taken at different stages of the bread-making process revealed that WUAX is degraded during the first fermentation, increasing WEAX. Additionally, the A/X ratio also increases, possibly due to the presence of xylanase in flour [25].

Furthermore, the structural changes and degradation of WUAX were also found during the brewing process of grain. The abundance of WEAX (WEAX yield) in brewers’ spent grain (consisting of 60% barley and 40% rice) is related to the enzymes and L. plantarum strains involved in the saccharification and fermentation processes. The average degree of polymerization (DP) of AX varied within a small range (4.7–6.3), but the average DP of the sample without saccharification and fermentation was as high as 15.6. In addition to its role in enhancing enzyme activity, analysis of the *L. plantarum* plant genome revealed the presence of genes encoding for hydrolases and esterases that contribute to the breakdown or utilization of AX [100]. Solid-state fermentation of brewers’ spent grain with *Fusarium oxysporum* f. sp. *lycopersici* showed significant differences in WEAX content, indicating the ability of this fungus to degrade WUAX. However, the content of WEAX decreases with extended fermentation time, likely due to the microorganisms using these polysaccharides for metabolism and growth [101]. 

Microbial fermentation is a natural and potentially gentler treatment method for modifying WUAX. Some microorganisms possess enzymes that can biodegrade WUAX, leading to changes in its structure and increased solubility, resulting in higher WEAX content. However, the use of this method is currently limited, and further research is needed to classify and study the microorganisms capable of degrading WUAX. Additionally, identifying the genes of the enzymes carried by these microorganisms could provide further insights.

In summary, the various methods of extracting WUAX have their own advantages and disadvantages. The cost of alkali extraction is lower, the operation is simple and the extraction rate is higher compared to other methods, whereas alkali extraction is more destructive to the structure of AX and the AX obtained may have a lower phenolic acid content. However, enzymatic extraction is selective for AX extraction, and the use of different enzymes and control of the amount of enzymes can produce AX with different structures, but the cost of enzymatic extraction is also relatively high. For the microbiological method, it is still an area to be developed, environmentally friendly and safe, and if it can be fully exploited and developed, it will be a promising method for application.

## 4. Technological Proprieties of Industrial Interest

### 4.1. Interaction with Gluten Protein

Gluten proteins have a decisive influence on the quality of flour products and are the main determinant of the viscoelastic properties of dough and final cereal products. The gluten network is formed after the hydration and mixing process of the dough and is stabilized through disulfide bonds and non-covalent bonds, such as hydrogen and ionic bonds [1]. Many studies have been carried out to understand the mechanisms of interaction between AX and gluten proteins. Wang et al. suggested that WUAX may act as a physical barrier, weakening the attraction between gluten molecules, directly interfering with the formation of gluten networks, and increasing the tensile strength of dough by altering the corresponding wheat gluten macromolecules [102]. Frederix et al. investigated the effects of WEAX and WUAX by adding their fractions with different MWs to wheat flour. The study of the effect on gluten adhesion in dough showed that WUAX was thought to harm gluten aggregates, reducing their numbers [15]. As discrete cell wall fragments, WUAX may interfere with gluten agglomeration by spatial dislocation or by creating physical barriers to interaction between gluten particles. Si et al. investigated the effect of interactions between glutenin and WUAX on the conformation of glutenin during heat treatment [103]. The results showed that the addition of WUAX reduced the viscoelasticity and thermal stability of gluten proteins, leading to a softening of the system. According to Wang et al., AX inhibits the formation of disulfide bonds in bread dough and interferes with protein aggregation. The low ferulic acid content of bran AX directly reduces dough extensibility as it is less cross-linked to gluten [1].

Given the negative effects of WUAX on gluten proteins in dough, many studies have been conducted to reduce these effects. Verjans et al. demonstrated that xylanase has the ability to degrade a certain amount of WUAX, with different types of xylanase presenting different abilities [104]. Jiang et al. added pentosidase (Pn) to the preparation of Chinese whole wheat steamed buns. The interaction of enzyme-interpreted AX with bran protein was investigated. The results showed that the addition of Pn reduced the MW of AX released from WUAX. The released AX increased the gluten content in the macromolecule, and the increase in Pn made the gluten network more porous and dispersed, which helped produce good dough extensibility. This indicates that the right amount of Pn is beneficial to the formation of a homogeneous fine-crumb structure, resulting in larger and less stiff whole-grain buns [105]. Sun et al. used enzymatic hydrolysis of WUAX to investigate the effects of WUAX and its hydrolysis products on the aggregation and structure of gluten proteins. The results showed that WUAX disrupted the original homogeneous network structure of gluten proteins and formed a dispersed network structure [106]. However, enzymatic degradation significantly reduced the average MW of WUAX and induced the aggregation of gluten protein molecules, and the resulting small molecule AX contributed to the formation of the gluten network structure, thus improving the quality of whole wheat products.

### 4.2. Effect on Physical Properties of Dough and Final Cereal Products

As the major non-starch polysaccharide in cereals, AX has various effects on many cereal-based products, such as bread, cakes, and doughnuts. AX plays an important role in dough and bread quality by interacting with other components of the flour and dough system [25]. In general, WEAX is usually beneficial for the quality of dough and bread as it increases water absorption, improves dough mixing stability, and increases bread volume, while the presence of WUAX has negative effects [107]. WUAX tends to disrupt the bubble interface of fermented doughs during bread making, thereby disrupting the pores [103]. Espinosa-Ramírez et al. compared the effect of WUAX extracted from maize on gluten-free dough at different levels of addition: in the presence of 1% AX, the dough matrix was observed to be inhomogeneous and lacking in gluten networks. The addition of 3% AX led to a complete change in this structure, forming a more homogeneous network [108]. Arif et al. investigated the effects of pentosan on the processing properties of durum wheat flour doughs and found that both water-extractable and water-unextractable pentosan had positive effects on dough performance by improving the water absorption capacity of flour, delaying dough maturation and improving stability as well as the dough mixing tolerance index [107]. However, the positive effect on bread parameters of adding water-extractable pentosan was greater. The water-holding capacity of WUAX in wheat bran was significantly higher than that of WEAX. A recent study has shown that bread with the addition of 10% AX retains more moisture after baking [109].

Based on the effect of WUAX on the properties of dough, it has been found that the addition of xylanase has a positive effect on dough quality. Yang et al. investigated the combined effect of xylanase (XYL) and glucose oxidase (GOX) in the production of whole wheat bread, which not only increased the gluten content of the dough (5.71%~13.04%) and the alcoholic protein solubility (3.57%~6.01%), but also reduced the viscoelasticity of the dough and resulted in a more open gluten skeleton structure [110]. The effects of XYL and GOX on wheat dough were highly dependent on the degree of WUAX hydrolysis. Xylanase likely converts part of the WUAX to WEAX. The molar mass distribution of either WEAX or WUAX in the wheat bran affects the strength of the dough, and this is related to the type of wheat [111,112]. The addition of high MW WUAX weakens the dough structure, whereas the low MW AX obtained after enzymatic digestion by xylanase eliminates this negative effect to some extent since the low MW AX forms a better gluten network and improves the stability of the dough [113]. However, current studies come to different conclusions on whether the A/X ratio of AX in bran affects the rheological properties of dough, which requires further investigation [1,111,112]. Overall, since the AX structure in cereal is greatly affected by the type and genotype of cereal, further studies should be carried out on certain specific varieties of cereal to maintain greater feasibility.

In cereals, WUAX is significantly more abundant than WEAX, and the MW and degree of substitution are relatively high. Therefore, WUAX is considered to be the predominant cause of the coarse texture and poor appearance of cereal products [114]. Crumb texture is one of the most important indicators of bread quality. WUAX has a high level of water absorption, which reduces the stability of the dough foam, ultimately reducing the volume of the bread and increasing the density of the bread matrix. As a result, the crumb hardness increases and the texture becomes rougher [109]. In a study by Espinosa-Ramírez et al., where WUAX from maize bran was added, there were no significant differences in crumb hardness between the breads from the pan-forming and the control breads, while the control breads had better elasticity, cohesion, and resilience. For the drop dough model bread, the bread with WUAX had lower hardness and chewiness as well as higher 2D area, height, cell density, and surface area compared to the control bread, especially at higher AX levels. However, AX has a negative effect on elasticity, cohesiveness, and resilience [108].

Specific volume is also an important bread quality parameter, and this is influenced by crumb structure, moisture content, and dough gas retention [109]. The effect of AX on bread volume is also highly dependent on fiber type and amount added. In a study by Koegelenberg and Chimphango, it was found that regardless of purity, WUAX obtained by alkali extraction at a dose of 0.8% replaced 2.5% of the flour without affecting the physical properties of the bread other than color, maintaining the weight, height, and volume of the bread [44]. However, Zhang et al. observed increased volume at all levels of adding bran AX, up to 5% [109]. The increase in specific volume after the addition of bran AX is due to the increase in strength and elasticity of the gluten-starch network [115].

To improve the quality of cereal products, Pihlajaniemi et al. added AX syrup, obtained by treating wheat bran with KOH, to bread, resulting in a softer texture [77]. Enzymes are widely used in the baking industry to improve the rheological and processing properties of doughs and the characteristics of the final product due to their greater efficacy and safety compared to chemical additives. Ghoshal et al. used partially purified xylanase in the production of whole-grain bread and investigated its effect on bread quality attributes at room temperature (25 ± 2 °C) and under refrigerated conditions (4 ± 1 °C). It reduced not only the water absorption of the dough but also the moisture loss of the bread during storage [116]. Arif et al. investigated the effect of pentosans on the quality of durum wheat bread and showed that the addition of aqueous pentosan increased bread volume and specific bread volume by 1–5%, whereas the addition of non-aqueous pentosan reduced bread volume by 30–44%. Both pentosans reduced crumb hardness by 5–20% [107]. Ghoshal et al. found that the addition of xylanase increased bread-specific volume, extended shelf life, reduced hardness during storage, reduced moisture loss, and improved bread color, and the addition of xylanase also improved the sensory properties of bread [116].

## 5. Biological Activities of AX from Gut Health Perspective 

### 5.1. Fermentability in the Human Gut

**Figure 3:**
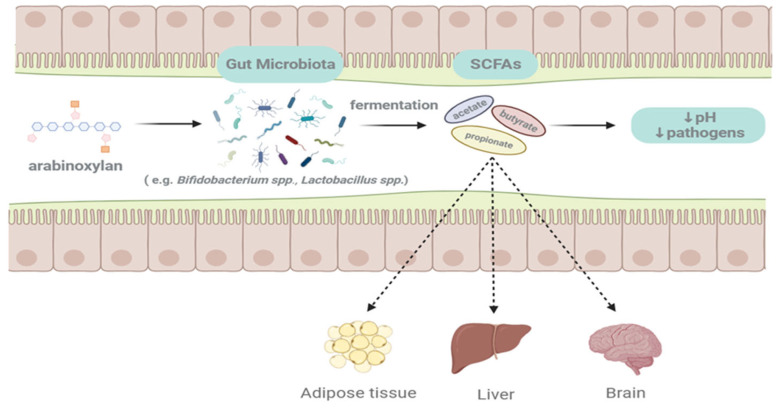
Arabinoxylan fermented in the human gut.

Due to its inability to be degraded by mammalian enzymes, AX can cross the small intestine and end up in the colon, where it is degraded by the gut microbiota. There are numerous sources of Axs, and the structure of AX obtained from different grains or different extraction methods varies, especially the carbohydrate structure of AX, in details including monosaccharide composition, branching, and average MW. These are important factors affecting its functional properties and may influence the fermentability and fermentation rate of AX in the human gut [1,3,16,22]. The diagram of AX fermenting in the gut is shown in Figure 3.

Since the prebiotic effects of AX can only be achieved through microbial fermentation, it is important to understand the types of microorganisms capable of utilizing AX. The most common microorganisms that utilize AXs are Bifidobacterium and Lactobacillus; other bacterial groups, such as Bacteroides, Prevotella, Roseburia, and Streptococcaceae can also be increased by AX. However, the carbohydrate structure of AX also influences the composition of the gut microbiota [22,117,118]. Table 3 summarizes the relevant microorganisms capable of fermenting AX from various sources.

The degree of fermentation of AX depends on its structure. Due to its complex structure, the fermentation rate in the human gut is slower, which means that WUAX stays in the human gut longer than WEAX. Unbranched AX has a higher fermentation ability than more-branched AX [119]. Damen et al. studied the intestinal fermentation of WUAX, WEAX, and AXOS in rats. WUAX was the most resistant to catabolism, with only 27.2% fermenting in the cecum and 42.7% catabolizing in the colon. The average arabinose substitution rate of diets containing WUAX increased from approximately 0.50–0.70 in the diet to 0.80–0.90, indicating that WUAX becomes more branched during fermentation [120]. Rose et al. investigated the fermentation of WUAX from maize, rice, and wheat bran in human feces and showed that AXs with high MW and monosubstituted arabinose side chains are able to sustain fermentation in the human intestine to produce short-chain fatty acids and that AXs can be degraded by different mechanisms depending on the diet [121]. Xu et al. used alkaline extraction to obtain CAX, then the extract was partially purified and hydrolyzed to analyze its structure and study the human intestinal flora fermentation, showing that highly branched and complex low-MW structures ferment more slowly in the human intestine, facilitating their transport to the colon and with fewer side effects [122]. Damen et al. have also shown that AX with a high MW or DP can have a positive effect on the rate of fermentation and the production of short-chain fatty acid (SCFA) [120].

In addition to the complex structure, the fermentation process and product of AX in the gut are also closely related to the type of grain and its genotype. Zhang et al. suggested that the gut microbiota is subjected to changes in the fine structure of fiber chemistry determined by plant genotypes and is not significantly influenced by the growth environment. Since AX extracted from corn bran of different genotypes has different branching structures, different changes are induced when fermented by the human gut microbiota, resulting in different SCFA synthesis [47]. Chen et al. observed that sorghum bran AXs fermented faster than CAX and that the branching structures of the two AXs differed, with AXs from sorghum bran having relatively uniform arabinose substitutions in the xylose backbone and AXs from corn bran having more complex glycan and chemical linkages [123]. In the results of the study by Yao et al. the more highly branched red sorghum bran AX produced more SCFA and several metabolites during colonic fermentation compared to white sorghum bran AX [124]. 

In general, AXs with low relative MW, low degrees of substitution, low FA content, and relatively simple structures have higher fermentation rates and less SCFA production, but due to the differences in AX structure and extraction process, the minor structural differences in AXs also strongly influence the metabolic results of fermentation, so it is difficult to fully discover the pattern of AX degradation and fermentation by the intestinal microbiota. However, different enzymes produced by different microorganisms in the gut have different abilities to degrade AX. Studies in this area are still lacking and tend to be at the microbial level, and the relationship between microbial species and the structure of AX could be explored in more detail.

**Table 3:** Gut microbial species affected by different sources of AX.

Material	Microorganism	A/X	Reference
Rice	*Akkermansia*	/	[125]
Wheat and sugar beet	*Bacteroides ovatus*	/	[118]
Sorghum	*Bacteroides*	0.96/0.83	[124]
Rye	*Bacterioides thetaiotaomicron*	0.62	[125]
Rye	*Bacteroides uniformis*	0.62	[125]
Rye	*Bacterioides vulgatus*	0.62	[125]
Corn bran	*Bifidobacterium longum*	0.56	[126]
sorghum	*Bifidobacterium*	0.96/0.83	[124]
Wheat	*Bifidobacterium animalis lactis*	/	[127]
Rye	*Bifidobacterium adolescentis*	0.62	[125]
Rye	*Clostridium beijerinckii*	0.62	[125]
Wheat bran	*Eubacterium rectale* spp.	0.63	[120]
Maize bran	*Lactobacilli*	0.72	[128]
Wheat	*Lactobacillus*	/	[129]
Corn bran	*Prevotella copri*	0.56	[126]
Wheat	*Roseburia* spp.	/	[127]
Wheat bran	*Roseburia*	0.63	[120]

### 5.2. Improvement of Gut Health

AXs are important members of the prebiotic family that targets beneficial microbial species in the gut, and their fermentation and degradation in the colon may affect SCFA production as well as the abundance and composition of the gut microbiota. Fermentation of AX significantly increased *Bifidobacterium* and *Lactobacillus* populations and decreased Enterobacteriaceae populations [128]. In addition to *Lactobacillus* and *Bifidobacterium* spp., the fermentation of AX in the gut can also increase other bacterial groups, including *Bacteroides*, *Prevotella*, *Roseburia*, and *Streptococcaceae*. Although these groups of bacteria are not considered to be the primary targets of prebiotics, they have also been shown to have a strong relationship with human health [117].

Li et al. used an in vitro fermentation model to simulate the fermentation of WUAX (RAX and CAX) and their deglycosylated counterparts (dRAX and dCAX) were extracted from rice bran and corn bran in the human gut and found that all types of AX significantly increased the relative abundance of *Bacteroides*, and that the most predominant SCFAs in the fermentation products after 48 h of fermentation were acetic and propionic acids, followed by butyric acid [32]. Likewise, Damen et al. evaluated gut microbiota composition and SCFA production in rats fed WUAX, WEAX, AXOS, and their combinations. Results showed that consumption of WUAX-enriched diets increased cecal and colonic butyrate content and production but did not stimulate cecal *Bifidobacterium* growth. An increase in bacteria, such as *Roseburia* and *Eubacterium rectale* spp., was also reported when rats were fed WUAX diets with higher DP compared to WEAX, resulting in an increase in butyric acid levels in the cecum of rats [120]. Neyrinck et al. treated mice with a control diet (CT), a high-fat diet (HF), or a high-fat diet supplemented with AX (10% *w*/*w*) for four weeks. The results showed that AX restored the number of bacteria reduced by HF feeding, i.e., species of the *Bacteroides-Prevotella* spp. and *Roseburia* spp., and that the addition of AX also significantly increased the content of *Bifidobacterium* in the cecum [127]. Nguyen et al. conducted a parallel two-group exploratory randomized controlled trial in 31 overweight and class I obese adults to investigate the composition of their fecal gut flora and SCFAs in the presence of high-dose long-chain AX supplementation. The results showed that AXs promoted specific intestinal flora classifications, including those associated with *Bifidobacterium longum*, *Blautia obeum*, and *Prevotella copri*, and further increased fecal propionic acid concentrations [126]. Truchado et al. investigated the modulatory effects of long-chain arabinoxylan (LC-AX) on the luminal and mucosal microbiota; the addition of 6 g/L LC-AX significantly increased the relative abundance of *Bifidobacterium* as well as propionic acid, among others, in the lumen and mucus compared to the control group [130].

**Figure 4:**
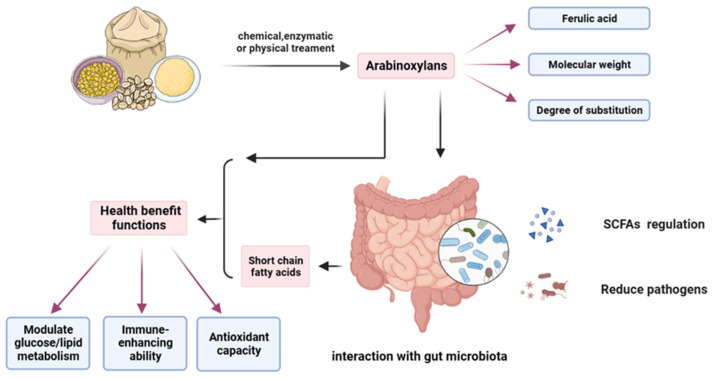
Effect on the human gut microbiota and potential health benefits of arabinoxylan from different cereals.

Differences in the structure and origin of WUAX not only affect their physicochemical properties but also have different effects on the gut microbiota. Overall, the fermentation of WUAX in the gut promotes the production of beneficial bacteria while increasing the production of SCFA. Also, due to its complex structure, such as its high DP, it is more effective than WEAX in interacting with gut microorganisms in some situations. The potential effects of AXs on host gut microbiota and health benefits are shown in Figure 4.

### 5.3. Alleviation of Metabolic Syndrome by Enhancement of Gut Health

The gut microbiota is a complex microbial ecosystem, and bacteria in the gut can ferment carbohydrates to produce substances such as SCFAs. These metabolites can act as a source of energy for colon cells and can increase feelings of fullness. Additionally, they have been shown to reduce inflammation, carcinogenesis, oxidative stress, and intestinal barrier function, all of which have a significant impact on human health [131,132]. As a typical dietary fiber, AX not only regulates the health of the human intestinal flora but also influences glucose and lipid metabolism and the occurrence of inflammation in the body.

Neyrinck et al. demonstrated that dietary supplementation with a water-extractable concentrate of high MW AX counteracted high-fat diet-induced dysbiosis of the gut ecology and improved adiposity and lipid-lowering effects. A significant increase in cecal *Bifidobacterium*, particularly *Bifidobacterium animalis*, was observed, and this effect was accompanied by improved gut barrier function and lower circulating inflammatory markers. More interestingly, rumenic acid was increased in white adipose tissue as a result of the AX treatment, suggesting that intestinal bacterial metabolism has an effect on host tissues. Thus, they suggest that AX may exert beneficial health effects by regulating the gut microbiota [127]. In another study by Chen et al., 50 male ICR/KM mice were randomly divided into five groups: Control diet (CON) group, High-fat diet (HFD) group, High-fat diet with AX (HFAX) group, High-fat diet with AX and β-glucan (HFAB), group and High-fat diet with AX and xyloglucan (HFAG) group. The results showed that compared to HFD, HFAB, and HFAG, mice had lower body weight, cholesterol, and triglyceride levels, reduced bile acids, increased microbial species diversity, increased numbers of beneficial bacteria, and reduced numbers of conditionally pathogenic bacteria [133]. Furthermore, research by Tong et al. showed that AX increased the production of SCFAs in the colon, leading to the excretion of lipids, cholesterol, and bile acids, ultimately reducing plasma total cholesterol and low-density lipoprotein (LDL) concentrations [134]. 

In addition, Luo et al. showed that mice supplemented with AX from rice bran (RAX) demonstrated significantly ameliorated obesity as induced by a high-fat diet, restoring intestinal microbiota alpha-diversity, increasing the relative abundance of anti-inflammatory bacteria such as *Bifidobacterium* and *Akkermansia*, and decreasing pro-inflammatory bacteria. These changes in the gut not only lead to a reduction in serum lipopolysaccharide (LPS) and systemic inflammation but ultimately to improved metabolic parameters and weight loss. The researchers believe that RAX is most likely able to restore host health by regulating the gut microbiome and its metabolite, SCFA [135]. Experimental results from Li et al. also support the possibility that AX may alter the colonic microbial metabolism by, for example, regulating intestinal flora and thereby improving the colonic mucosal barrier. After feeding rats diets containing AX for 5 weeks, the number of *E. coli* in the colonic digestive fluid decreased, while the number of *Lactobacillus*, *Bifidobacterium*, and *Bacteroides* increased, and serum lipopolysaccharides decreased in rats. Furthermore, AX upregulated the levels of genes for proteins associated with tight junctions in the colonic mucosa [129].

In conclusion, both WUAX and WEAX have potential health benefits, but their functional activity is mainly influenced by their structural characteristics and the source of the grain. The structural characteristics of AXs may alter the interaction of AXs with the gut microbiome, thereby affecting SCFA production and its effects on the host. AX has been shown to regulate the gut microbiome and its metabolites, leading to improvements in human health. However, the exact mechanism of action and its effects on gene expression relating to glucose and lipid metabolism are still unclear. It can be assumed that some of the effects of AXs on human health are indeed achieved by regulating the gut microbiome and its metabolites.

## 6. Conclusions and Perspective

The structure of Axs is influenced by factors such as source, maturity, grain location, extraction and purification methods, and specific processing techniques. While WEAX has been extensively studied and shown to possess numerous beneficial properties, its low content in cereals poses challenges for industrial production. On the other hand, WUAX has high content and availability, offering great potential for exploration. The chemical structure of AX greatly impacts the physical properties of food. Modifying WUAX in various ways can positively influence the food matrix. Additionally, WUAX has potential health benefits, including modulation of SCFA in the colon, improved antioxidant capacity, and reduced blood glucose response through various mechanisms. Therefore, future research must focus on understanding the structure–activity relationship of WUAX. This involves investigating the correlation between the structure and function of AXs, comparing their activities in the same test system, and establishing specific principles. To enhance the utilization of WUAX, it is essential to develop gentle and harmless methods, such as microbial fermentation, to obtain AXs with specific structures. This would result in the release of more WUAX. In addition, understanding the relevant gut microbial species capable of fermenting and using AXs and the enzyme systems they produce is important for better utilization of AXs of different structures. By utilizing WUAX more effectively in food production, both the quality of food products and human health can be improved.

## Figures and Tables

**Table 1:** Effect of alkali extraction on the A/X ratio and MW of WUAX in different grains.

Source	Extraction	MW/kDa	A/X	Yield (%)	Reference
Wheat bran	NaOH	351.7	0.83	18.5	[69]
Wheat bran	NaOH and Ca(OH)_2_	437	1.14	-	[70]
Wheat bran	NaOH	694	0.95	6.5	[51]
Wheat bran	NaOH	711	0.51	-	[63]
Wheat bran	NaOH	163	0.77	-	[71]
Barley bran	NaOH	298.36	0.58	5.31	[46]
Barley Bran	NaOH	318.2	0.55	8.42	[72]
Barley endosperm	NaOH	758.2	0.76	2.13	[72]
Peeled barley seeds	Ba(OH)_2_	1360	0.60	1.6	[73]
Sorghum seeds	Ca(OH)_2_	223.9	1.09	17 ± 2.40	[74]
Red sorghum bran	NaOH	356	1.1	6.41 ± 0.27	[75]
White sorghum bran	NaOH	136.2	1.08	7.14 ± 0.12	[75]
Oat grain	NaOH	100	0.11	8	[42]
Corn bran	NaOH	360	0.54	-	[16]
Corn bran	NaOH	770	0.51	-	[63]
Corn bran	NaOH and Ca(OH)_2_	233.3	0.76	17.5 ± 0.2	[76]
Rye bran	Ba(OH)_2_	381	0.6	5.84	[41]
Rye bran	NaOH and Ca(OH)_2_	234.9	0.72	11.5 ± 0.4	[76]

**Table 2:** Effect of enzyme extraction on the A/X ratio and MW of WUAX in different cereals.

Material	Enzyme	Origin	MW/kDa	A/X	Reference
Wheat bran	Xylanase	*Trichoderma reesei*	32.52	0.56	[69]
Wheat bran	Endo-xylanase	*Geobacillus stearothermophilus*	/	0.44	[82]
Wheat bran	Endo-xylanase	*Thermomyces lanuginosus* (donor)/*Aspergillus oryzae* (host)	1–25 (85.7%) 25–700 (14.3%)	0.81	[93]
Wheat bran	Endo-xylanase	*Bacillus subtilis*	12.5	0.83	[80]
Wheat bran	Endo-xylanase	Multifect^®^ CX XL	420	1.14	[16]
Corn bran	Xylanase	Multifect^®^ GC-extra	51.6–132	/	[92]
Corn bran	Endo-xylanase	Multifect^®^ CX XL	43	0.54	[16]
Rice bran	Endo-xylanase	Multifect^®^ CX XL	23	0.97	[16]
Rice bran	Xylanase	*Bacillus subtilis*	57.9	0.29	[94]
Rice bran	Xylanase	*Bacillus subtilis*	41.4	0.3	[94]

## Data Availability

No new data were created or analyzed in this study. Data sharing is not applicable to this article.
